# Molybdenum and Cadmium Co-induce Mitochondrial Quality Control Disorder *via* FUNDC1-Mediated Mitophagy in Sheep Kidney

**DOI:** 10.3389/fvets.2022.842259

**Published:** 2022-01-28

**Authors:** Yunhui Wu, Fan Yang, Guangbin Zhou, Qi Wang, Chenghong Xing, He Bai, Xin Yi, Zhiwei Xiong, Shuqiu Yang, Huabin Cao

**Affiliations:** ^1^Jiangxi Provincial Key Laboratory for Animal Health, Institute of Animal Population Health, College of Animal Science and Technology, Jiangxi Agricultural University, Nanchang, China; ^2^Animal Epidemic Prevention and Quarantine Unit, Fengcheng Agricultural and Rural Bureau, Fengcheng, China; ^3^Animal and Plant Quarantine Office, Nanchang Customs, Nanchang, China

**Keywords:** molybdenum, cadmium, mitophagy, mitochondrial quality control, sheep, kidney

## Abstract

Molybdenum (Mo), fundamental trace mineral for animals and plants, but undue Mo damages animal health. Cadmium (Cd) is a toxic heavy metal that exists in the environment. Nevertheless, the mechanism of Mo and Cd on mitochondrial quality control are still indistinct. The objective of this research was to explore the effects of mitophagy on mitochondrial quality control via the FUNDC1-mediated by Mo and Cd in sheep kidney. Forty-eight 2-month-old sheep were stochastically divided into four groups, as shown below: control group, Mo [45 mg/kg body weight (BW)] group, Cd (1 mg/kg BW) group and Mo (45 mg/kg BW)+Cd (1 mg/kg BW) group, with 50 days feed technique. The results showed that Mo or/and Cd attract an unbalance of trace minerals and vacuoles and granular degeneration of renal tubular epithelial cells, and increase the number of mitophagosomes and vacuole-mitochondria and LC3 puncta and MDA and H_2_O_2_ contents, and decrease ATP content in the kidney. Moreover, Mo or/and Cd treatment could upregulate the mRNA levels of FUNDC1, LC3A, LC3B, PGAM5, DRP1, FIS1 and MFF, and the protein levels of FUNDC1, p-FUNDC1, LC3II/LC3I, DRP1, MFF and FIS1, downregulate the mRNA levels of MFN1, MFN2, OPA1, PGC-1α, SIRT1, SIRT3, FOXO1 and FOXO3, and the protein levels of MFN1, MFN2, OPA1 and PGC-1α. Notably, variations of above-mentioned factors in Mo and Cd group were more obvious than in Mo or Cd groups. Taken together, these results displayed that Mo and Cd co-treatment might induce mitochondrial quality control disorder via FUNDC1-mediated mitophagy in sheep kidney.

## Introduction

Molybdenum (Mo), a fundamental trace minerals for animals, plants and most microorganisms, primarily occurs in multitudinous metalloenzymes (1). It is a part of the active site of metalloenzyme and is usually used to catalyze various redox reactions (2). For one thing, the lack of Mo is fatal to organisms. For another, undue Mo could lead to toxic symptoms, that is, organisms only need a small amount of Mo (2). Importantly, ruminants are more impressionable to high level of Mo than other kinds of animals (3, 4). Furthermore, Mo is also widely used in industry, especially metal alloys, pigments, lubricants and fertilizer manufacture, and due to the rapid development of modern industry, the content of Mo in the environment is gradually accumulating (5, 6). The kidney are the main short-term storage organs of Mo, and molybdate is excreted through urine (7). Previous researches have revealed that undue Mo could cause damage to renal tissue, such as degeneration of renal tubule and glomerular atrophy, degrees of vacuolization, irregularity, fission of the nucleus, and swelling of mitochondria (4, 8). However, the specific mechanism of mitochondrial damage caused by Mo still needs to be explored.

Cadmium (Cd) is a poisonous heavy metal (9), which exists in the environment and is classified as a Group 1 human carcinogen (10). It is widely used in electronics, metallurgy, pigments and plastic stabilizers (11). Cd has a long biological half-life period, which can enter animals and humans through food cycle and enrich in the body for long periods, causing injury to the internal organs (12, 13). The kidney is a crucial target organ of Cd-accumulation (14). It is interesting to note that mitochondria are the key intrastitial target spots of Cd-mediated cytotoxicity (15). A large number of studies have revealed that Cd induces mitophagy via the PINK/Parkin pathway (16–18). However, the mechanism of Cd on mitochondria needs further study.

Mitochondria have evolved elaborate quality control systems to adapt to and respond to complex external pressures and to ensure the necessary number of functional mitochondria (19). The process is controlled by two opposing forces, which include mitochondrial dynamics, mitochondrial biogenesis to compensate for mitochondrial function, and mitophagy to degrade damaged mitochondria (20). Mitochondrial dynamics refers to the process in which mitochondria maintain their dynamic balance through fusion and fission (21), which are crucial quality control mechanisms for mitochondrial preservation (22). Mitochondrial biogenesis is an intricate biological process, which controls the self-renewal of mitochondria and the maintenance of mtDNA, thus maintaining intracellular homeostasis (23). Mitophagy is a major pathway by which eukaryotic cells remove damaged or no longer needed mitochondria under a variety of physiological and pathological conditions (24). Under pressure, the damaged mitochondria are encapsulated in isolation membranes, membranes seal and fuse with the lysosomes to eliminate damaged mitochondria (17). The functional state of mitochondria depends on the dynamic balance among the biogenesis, fusion, division and autophagy of mitochondria (25). The PTEN-induced putative protein kinase 1 (PINK1)/Parkin pathway is one of the well-known mitophagy pathways. In addition to the PINK1/Parkin pathway, the other pathway depends on a group of receptor proteins, such as FUNDC1 and BCL2 interacting protein 3 (BNIP3)/a homolog of the E1B 19K/Bcl-2 binding and pro-apoptotic protein Nip3 (Nix) (26). Studies have shown that Mo or Cd could induce mitochondrial damage (27, 28). However, whether the disorder of mitochondrial quality control system is the underlying mechanism of mitochondrial damage by Mo or/and Cd deserves further study.

Since most tungsten ores in Gannan, Jiangxi Province belong to the associated minerals of Mo, Cd and wolfram (W), a large number of tailings and tailings water discharged during the development of W ores contain heavy metal elements such as Mo and Cd. Heavy metals such as Mo and Cd can be deposited in the soil, which could be absorbed and enriched by plants and migrate through the food chain, causing poisoning of livestock and poultry by Mo and Cd, thus seriously threatening the healthy development of animal husbandry in this area and the food hygiene and safety of residents (29). At the same time, ruminants are extremely sensitive to Mo, and it is of great significance to study the combined toxicity of Mo and Cd to ruminants. Currently, sheep have become an important economic animal in livestock breeding in Jiangxi Ganan, and are also one of the main livestock at risk of Mo and Cd pollution in the local area. However, most studies focus on single heavy metal exposure, while in nature, humans and animals are often exposed to various heavy metals. Therefore, this study was to search for the mechanism of Mo and Cd co-induce mitochondrial quality control disorder via constructing the experimental models of sheep with the treatment of Mo or/and Cd and provided a theoretical basis for toxicological exploration.

## Materials and Methods

### Animals and Treatments

All animals in this experiment were approved by the Committee of Animal Welfare (NO. JXAULL-2020-04). Animal care and experimental procedures were also complied with the criteria of the Institutional Animal Care and Use Committee Guidelines at College of Animal Science and Technology, Jiangxi Agricultural University.

All the sheep were raised in the same conditions during the experiment, which was fed elephant grass [dry matter (DM)] with supplementary fodder at 08:00 am and 3:00 PM each day, and were allowed drinking water adlibitum. The composition and nutrient levels of the basal diet of sheep are shown in Supplementary Table 1. In this study, the sheep were treated with (NH_4_)_6_Mo_7_O_24_4H_2_O and/or CdCl_2_. The group was given normal saline by gavage, while the experimental groups were given corresponding drugs by gavage for 50 consecutive days. A total of 48 two-month-old healthy sheep were stochastically divided into four groups, as shown below: control group; Mo (45 mg Mo·kg^−1^·BW) group; Cd (1 mg Cd·kg^−1^·BW) group; Mo + Cd (45 mg Mo·kg^−1^·BW+1 mg Cd·kg^−1^·BW) group. The dosage of CdCl_2_ and (NH_4_)_6_Mo_7_O_24_4H_2_O were selected from previous studies (30). Ammonium molybdate tetrahydrate (NH_4_)_6_Mo_7_O_24_4H_2_O and cadmium chloride (CdCl_2_) were supplied by Shanghai Macklin Biochemical Co., Ltd, China.

### Sample Collection

On the 25 day of the experiment, twenty-four sheep were randomly drawn and killed with an intravenous overdose of pentobarbital. The kidneys of each sheep were then removed, immediately frozen in liquid nitrogen and finally stored at−80°C. The sample was used for RNA extraction. On day 50, the remaining sheep were killed using the same method. Fresh kidney tissue was removed, part was placed in electron microscope solution and stored at 4°C, part was placed in section solution and stored at room temperature, and the rest was placed in cryopreservation tubes and immediately placed in liquid nitrogen and stored at -80°C.

### Detection of Trace Minerals

The contents of Mo, Cd, copper (Cu) and zinc (Zn) in tissues were determined by graphite digestion. About 200 mg of kidney tissue was taken, and it was put into the matching digestive tract, 65% hydrochloric acid and 65% nitric acid were added, and it was made into a 6 mL mixture. After cooling, the remaining liquid was attenuated to 10 mL with soft water, and spotted by inductively coupled plasma mass spectrometry (ICP-MS) equipment.

### Histopathological and Ultrastructural Assessment

Fresh kidney tissues were collected, fixed in 4% paraformaldehyde, dehydrated with ethanol gradient. Next, ethanol was removed with xylol, and tissues were implanted in paraffinum, sectioned and colored with hematoxylin and eosin (H&E). The histomorphology of the kidney were scanned under a microscope (Olumpus, Japan). The ultrastructural observation was implemented based on the step as previously described (31). The morphological structure of mitochondria were scanned under microscope transmission electron microscopy (Zeiss, Germany).

### Immunofluorescence Co-location Analysis

Fresh kidney tissues were fixed with 4% formaldehyde, and they were sectioned. Then permeabilized with 0.2% of Tween-20 at room temperature for 10 min. Sections were incubated with antibodies to LC3 and COX IV (1:200, dilution) followed by fluorescently labeled secondary antibodies and DAPI. The expression and location of COX IV and LC3B were captured using a Leica TCS SP8 confocal microscope.

### Oxidative Stress Level Determination

The kidney tissue weight was accurately weighed and then mixed with 9 times the volume of 0.9% NaCl. Homogenates were obtained under ice-water bath conditions, and 10% kidney tissue homogenates were obtained. Then the homogenate was centrifuged for 10 min at 2,500 revolutions per minute (rpm), and the supernatant was removed. The activities or contents of malondialdehyde (MDA), hydrogen peroxide (H_2_O_2_), catalase (CAT), superoxide dismutase (SOD), glutathione peroxidase (GSH-Px) were examined using commercial kits provided by the Jiancheng Biological Engineering Research Institute, Nanjing, China. All tests were carried out based on the manufacturer's instructions. In short, the content of H_2_O_2_ and MDA and the activities of CAT, GSH-px and SOD could be calculated by their specific formulae by measuring their absorbance at a certain wavelength on a microplate reader (SpectraMax M2, US).

### ATP Content Determination

The adenosine triphosphate (ATP) was examined using commercial kits provided by the Jiancheng Biological Engineering Research Institute, Nanjing, China. The test was carried out based on the manufacturer's instructions. 0.1g of fresh kidney tissue were homogenized using alkaline lysis buffer. Then the lysate was centrifuged for 10 minutes at 12,000 rpm. The supernatant was collected and the protein concentration was detected using a protein assay kit. The sample and the ATP test reagent were mixed and stood for 5 min, and the absorbance value was determined at 636nm wavelength.

### Real-Time Quantitative Polymerase Chain Reaction (RT-qPCR) Analysis

The specific experimental method of RT-QPCR was referred to a previous study (32). TRIzol reagent (Vazyme Biotech, China) was used to segregate total RNA from kidney tissue following the explanations supplied by the manufacturer. The concentration of RNA was measured using a nucleic acid protein analyzer. A PrimeScript RT reagent kit (Vazyme Biotech, China) was used to inverse transcribe complementary DNA (cDNA). The cDNA were deposited at −20°C for RT-qPCR. The cDNA and reaction system were mixed together and then added to the 96-well plate. The mRNA levels of related genes were detected using fluorescence quantitative PCR (QuantStudio 7 Flex). The mean values of GAPDH levels were normalized and the relative changes in mRNA levels were computed using the 2^−ΔΔCT^ method. The primers used for RT-qPCR analysis are exhibited in Table 1.

**Table 1:** Premier sequences used for real-time PCR.

**Gene**	**5'-Primer (F)**	**3'-Primer (R)**
FUNDC1	CTGGTGGAATCGCGTGTTTG	CCACTGTGACTGGCAATCTGA
PGAM5	CCGGAGGCTGTGCAGTATTA	TTGGCGTGGCAGATGAAGAT
LC3A	CCAGGAAACCTTCGGCTTCT	AGAGGCAGAGGAGGTGTAGT
LC3B	CATGAGCGAGTTGGTCAAGA	GATGTCAGCGATGGGTGTGG
DRP1	GCTGGAGAAGAGGAATCGCAT	GCAGCCATTTCAAAACCCAGG
FIS1	AGGCCATGAAGAAAGATGGACT	GACGTTCACAGAGGACAGGG
MFF	GGCGTTCTCGCGTCTCTCTT	TTTAGAGCCACTTTTGTCCCCCTG
OPA1	AAGAATCGGACCCAAGAGCA	CTTCCACTCCTCGAGACTCC
MFN1	CTTTTGAGGAGTGTATCTCGCAG	GTCCAGTATGTTTTTCACAGTGTCT
MFN2	GGAAGCCCTGGCGATGAATA	CCAGTTCCGAGTTACTTCACCT
PGC-1α	GAAAGGGCCAAACAGAGAGA	GTAAATCACACGGCGCTCTT
SIRT1	TGCTCGCCTTGCAATAGACT	TCCACTGCACAGGCCACATAC
SIRT3	GAATGGCGCTGTTTCCTTGTT	CAACCCCTGGAGACCTGAAGT
FOXO1	TATCGAGCGCTTGGACTGTG	TTTAAGTGTAGCCTGCTCGC
FOXO3	TGTTGGTTTGAACGTGGGGA	CGTCAGTTTGAGGGTCTGCT
GAPDH	TGATGCTCCCATGTTCGTGA	CTTTTCCCACAGCCTTAGCAG

*F, Forward; R, Reverse*.

### Western Blotting

Approximately 0.1 g kidneys were taken from each sheep and total proteins were extracted with a mixture containing 1 mM phenylmethanesulfonyl fluoride (PMSF) and RIPA lysis buffer (Solarbio, China), and the concentration of albumen was tested by bicinchoninic acid (BCA) protein kit (Solarbio, China). The protein was separated by sodium dodecyl sulfate-polyacrylamide gel electrophoresis (SDS-PAGE), then transferred to polyvinylidene fluoride (PVDF) membranes (Biosharp, China). The PVDF membrane was blocked with phosphate buffer saline tween (PBST) containing 5% fat-free milk for 1 h at indoor temperature, the membrane was incubated overnight with diluted primary antibodies against FUN14 domain-containing 1 (FUNDC1) (Affinity, China), p-FUNDC1 (Ser17) (Affinity, China) microtubule-associated protein 1 light chain (LC3B) (Wanleibio, China), optic atrophy 1 (OPA1) (Bioss, China), mitofusin 1 (MFN1) (Proteintech, USA), mitofusin 2 (MFN2) (Proteintech, USA), dynamin-related protein 1 (DRP1) (Wanleibio, China), mitochondrial adaptor fission 1 (FIS1) (Proteintech, USA), mitochondrial fission factor (MFF) (Proteintech, USA), peroxisome proliferator-activated receptor γ coactivator 1α (PGC-1α) (Wanleibio, China) and GAPDH (Bioss, China), followed by the corresponding HRP-conjugated secondary antibodies (Bioss, China). The signal blots were tested with electrochemiluminescence liquid (ECL) (Vazyme Biotech, China). Then the gray value of the matching protein was measured by Image J software.

### Statistical Analysis

GraphPad Prism 8.0 (GraphPad Inc., LaJolla, CA, USA), the SPSS 25.0 (SPSS Inc, Chicago, IL, USA) were used to analyze the data. The data were analyzed statistically between the control group and treated groups of sheep by one-way analysis of variance (ANOVA). The significance of intergroup differences was analyzed by using least-significant difference (LSD). All data are described as mean ± standard deviation (SD), and a *P*-value < 0.05 was deemed to indicate statistical significance.

## Results

### Effects of Mo and/or Cd on Trace Elements and Histopathology in the Kidney

**Figure 1:**
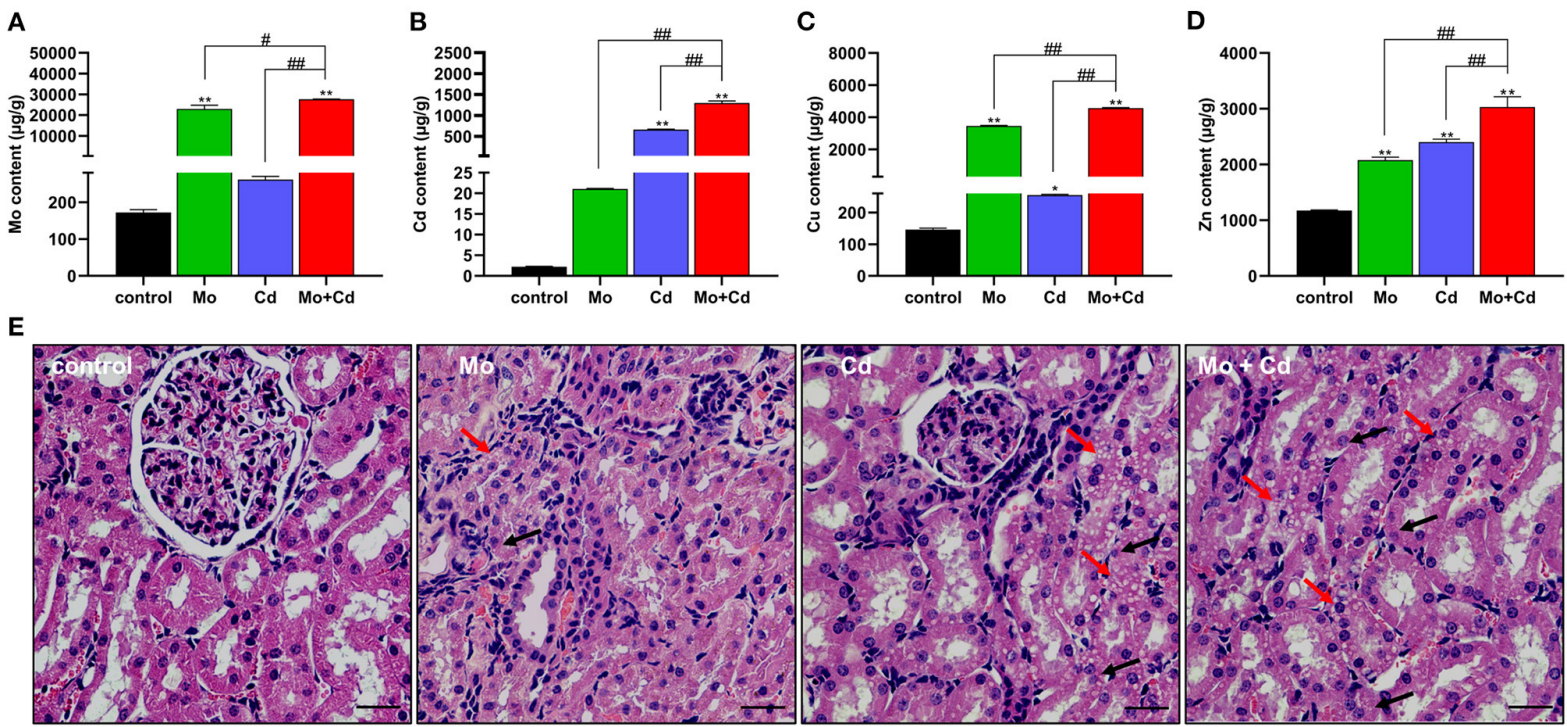
Effects of Mo and/or Cd on trace elements and histopathology in the kidney. **(A)** Mo content, **(B)** Cd content **(C)** Cu content **(D)** Zn content. **(E)** Histological characteristics of the kidney. The histopathological lesions included the vacuolar degeneration (red arrows) and granular degeneration (black arrows) in the renal tubular epitheliums. HE staining, scale bar = 50 μm. Data are expressed as means ± SD (*n* = 6). “*” indicates significant difference compared to the corresponding control (*P < 0.05, **P < 0.01). “#” indicates statistically significant difference between corresponding groups (^#^*P* < 0.05, ^*##*^*P* < 0.01).

The data of Mo, Cd, Cu and Zn in each group were determined as shown in Figures 1A–D. After being treated with Mo and/or Cd in the feed for 50 days, the content of Mo was significantly aggrandized (*P* < 0.01) in Mo and Mo + Cd groups and the Cd content was markedly increased (*P* < 0.01) in Cd and Mo + Cd groups in comparision with the control group. The contents of Cu and Zn were significantly increased (*P* < 0.05 or *P* < 0.01) in three treatment groups compared with the control group. In addition, the Mo, Cd, Cu and Zn contents in Mo + Cd group were dramatically higher than those in Mo and Cd groups (*P* < 0.05 or *P* < 0.01). Tissue slice of the kidney in all groups were appeared in Figure 1E. In the control group, normal renal histology was observed, with had smooth round nuclei. Treatment with Mo and/or Cd can cause histopathological changes of the kidney, including vacuoles and granular degeneration of renal tubular epithelial cells.

### Mo and/or Cd Co-induced Oxidative Stress in Kidney of Sheep

**Figure 2:**
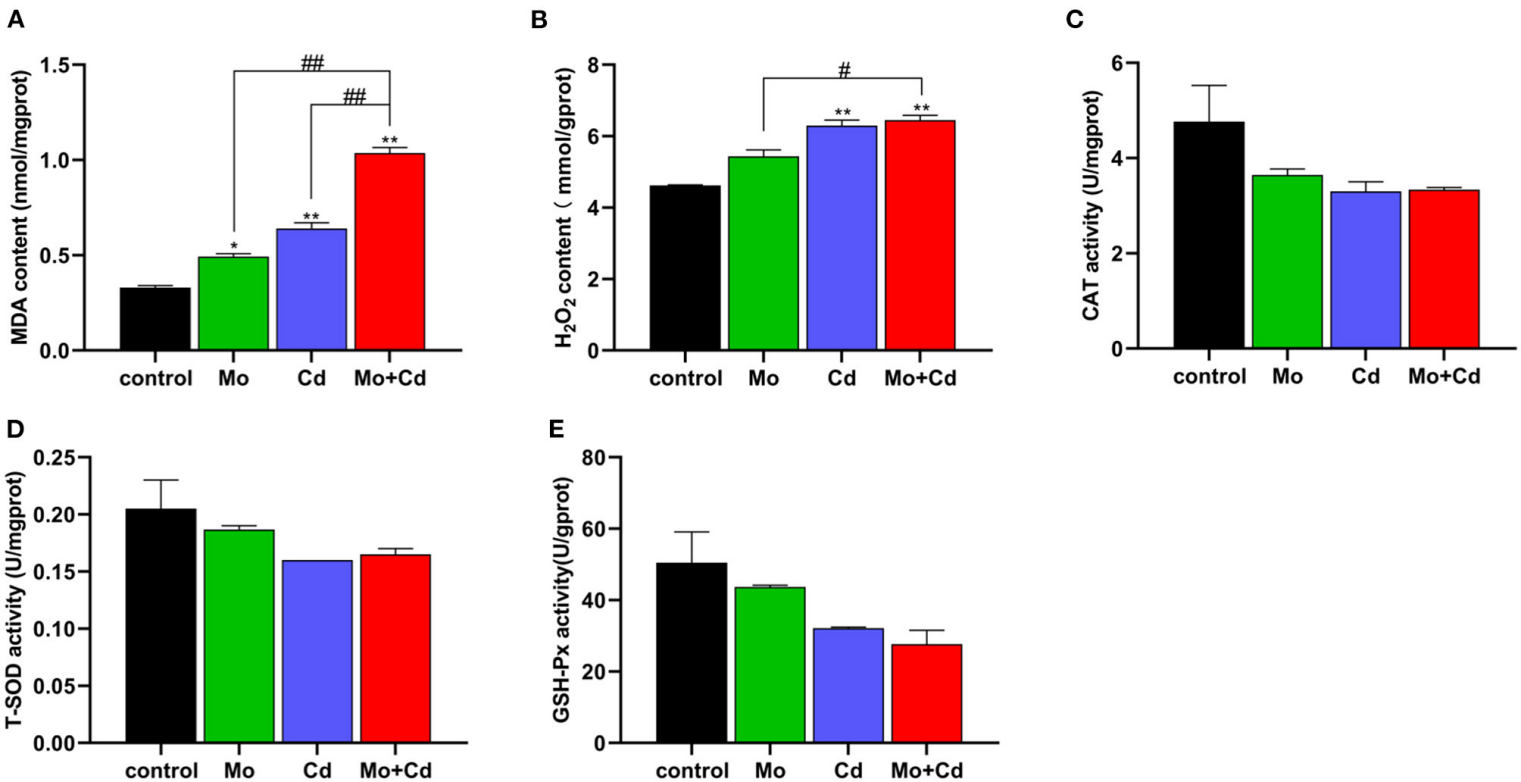
Effect of Mo or/and Cd on oxidative stress of the kidney. **(A)** MDA content, **(B)** H_2_O_2_ content, **(C)** CAT activity, **(D)** T-SOD activity, **(E)** GSH-Px activity. Data are expressed as means ± SD (*n* = 6). “*” indicates significant difference compared to the corresponding control (*P < 0.05, **P < 0.01). “#” indicates statistically significant difference between corresponding groups (^#^*P* < 0.05, ^*##*^*P* < 0.01).

We determined oxidative stress level at day 50. The content of MDA was significantly increased (*P* < 0.05 or *P* < 0.01) under Mo or/and Cd coerce (Figure 2A), and H_2_O_2_ content in Cd and Mo + Cd groups significantly aggrandized (*P* < 0.01) in comparison with the control group (Figure 2B).The contents of MDA and H_2_O_2_ in Mo + Cd group were notably higher than those in Mo or Cd groups (*P* < 0.05 or *P* < 0.01). In addition, T-SOD, CAT and GSH-Px activities in experimental groups were all decreased (*P* > 0.05) in comparison with the control group (Figures 2C–E).

### Effect of Mo or/and Cd on Ultrastructure and ATP Content of the Kidney

**Figure 3:**
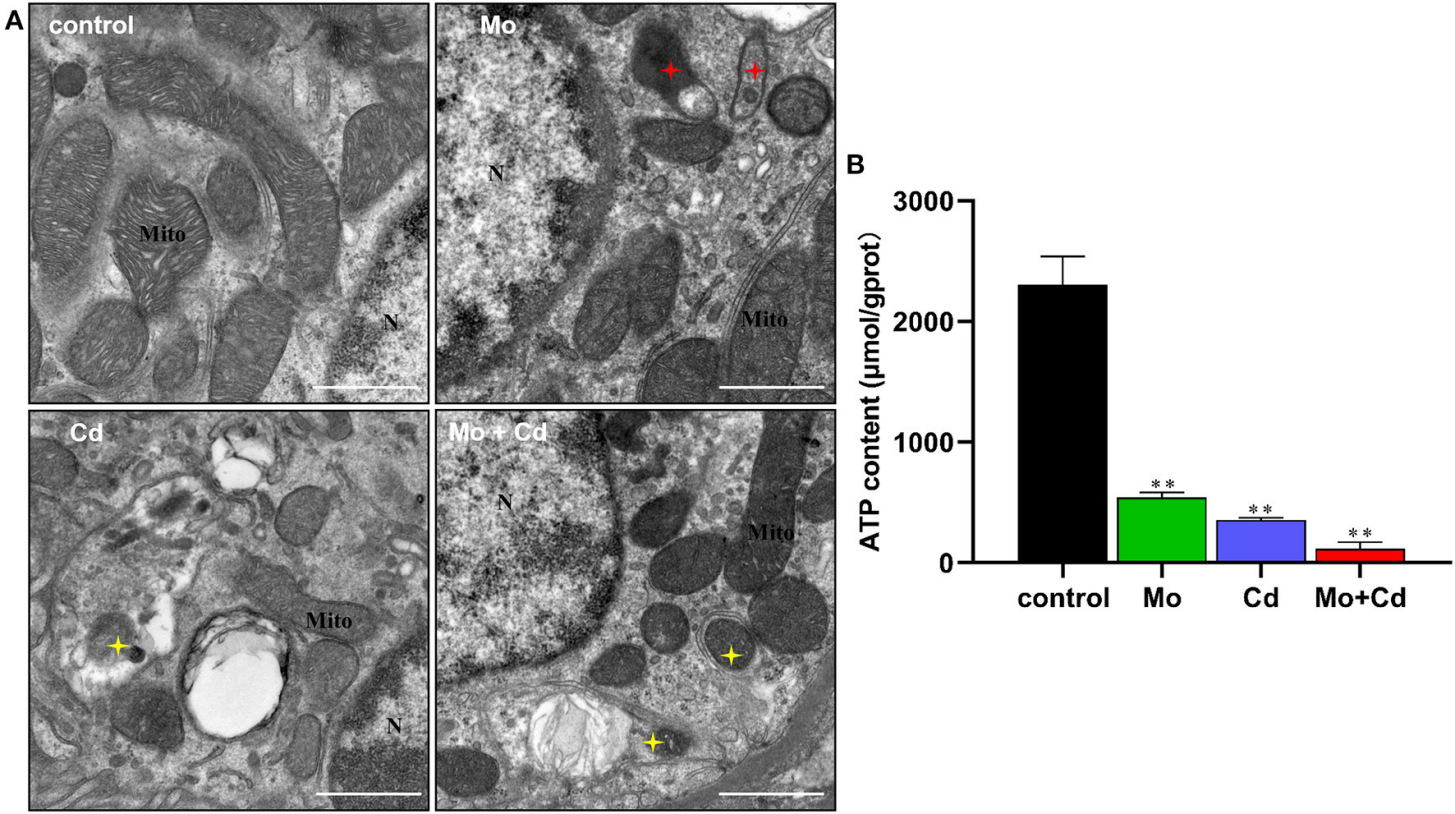
Effect of Mo or/and Cd on ATP level and ultrastructure of the kidney. **(A)** The ultrastructural structure of the kidney. Red asterisks mark vacuole-mitochondria. Yellow asterisks indicate mitophagosomes. “N” means nucleus, “Mite” means mitochondrion, scale bar = 1 μm. **(B)** ATP content. Data are expressed as means ± SD (*n* = 6). “*” indicates significant difference compared to the corresponding control (*P < 0.05, **P < 0.01). “#” indicates statistically significant difference between corresponding groups (^#^*P* < 0.05, ^*##*^*P* < 0.01).

The results of ultrastructural observation are shown in Figure 3A. The TEM observations showed that the mitophagosomes and vacuole-mitochondria numbers increased in renal tissue following Mo or/and Cd exposure. We determined ATP content at day 50 using an ATP determination kit. Mo or/and Cd treatment significantly reduced (*P* < 0.01) ATP content compared with the control group (Figure 3B).

### Mo or/and Cd Exposure Regulated Mitochondrial Fusion and Fission

**Figure 4:**
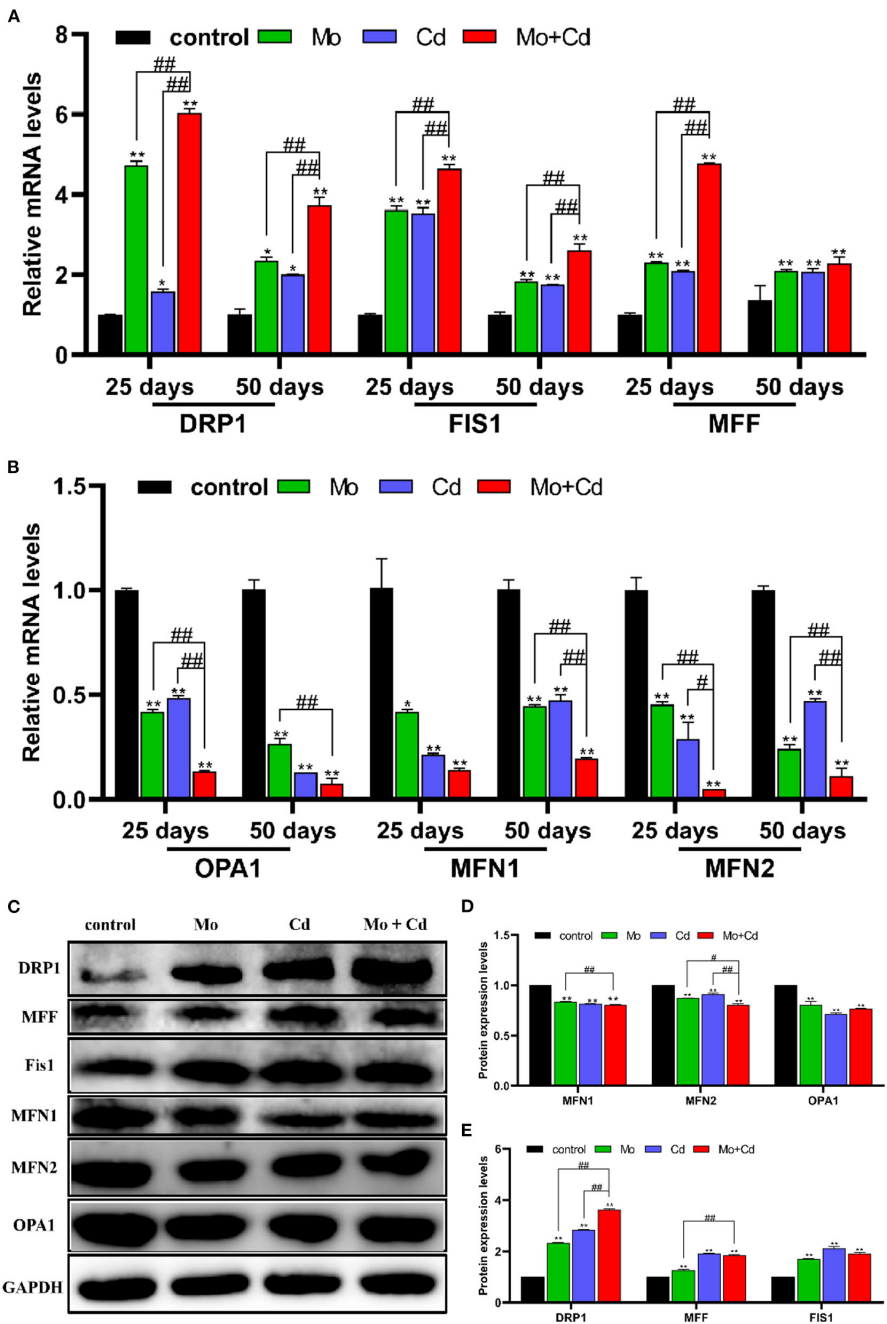
Mo or/and Cd exposure regulated Mitochondrial fusion and fission (mitochondrial dynamics). **(A)** The mRNA levels of DRP1, MFF and FIS1, **(B)** The mRNA levels of MFN1, OPA1 and MFN2. **(C)** The western blot results of DRP1, MFF, FIS1, MFN1, OPA1 and MFN2. **(D,E)** The quantification of DRP1, MFF, FIS1, MFN1, MFN2, OPA1 and PGC-1α protein levels. Data are expressed as means ± SD (*n* = 6). “*” indicates significant difference compared to the corresponding control (*P < 0.05, **P < 0.01). “#” indicates statistically significant difference between corresponding groups (^#^*P* < 0.05, ^*##*^*P* < 0.01).

To explore the outcomes of Mo and/or Cd exposure on mitochondrial dynamics in the kidney, we spotted the mRNA and protein levels of mitochondrial dynamics-related at days 25 and 50. As shown in Figures 4A,B, Mo or/and Cd coerce significantly aggrandized (*P* < 0.05 or *P* < 0.01) the mRNA levels of DRP1, MFF and FIS1, and significantly lessened (*P* < 0.05 or *P* < 0.01) the mRNA levels of MFN1, MFN2 and OPA1 at days 25 and 50. In addition, we found that the protein levels of DRP1, MFF and FIS1 were increased (*P* < 0.05 or *P* < 0.01) significantly, and the protein levels of MFN1, MFN2 and OPA1 were decreased (*P* < 0.05 or *P* < 0.01) significantly at day 50 under Mo or/and Cd coerce (Figures 4C–E). In addition, the variation of these above factors in Mo and Cd co-treated groups were more evident than those in Mo or Cd groups.

### Mo or/and Cd Exposure Regulated Mitochondrial Biogenesis

**Figure 5:**
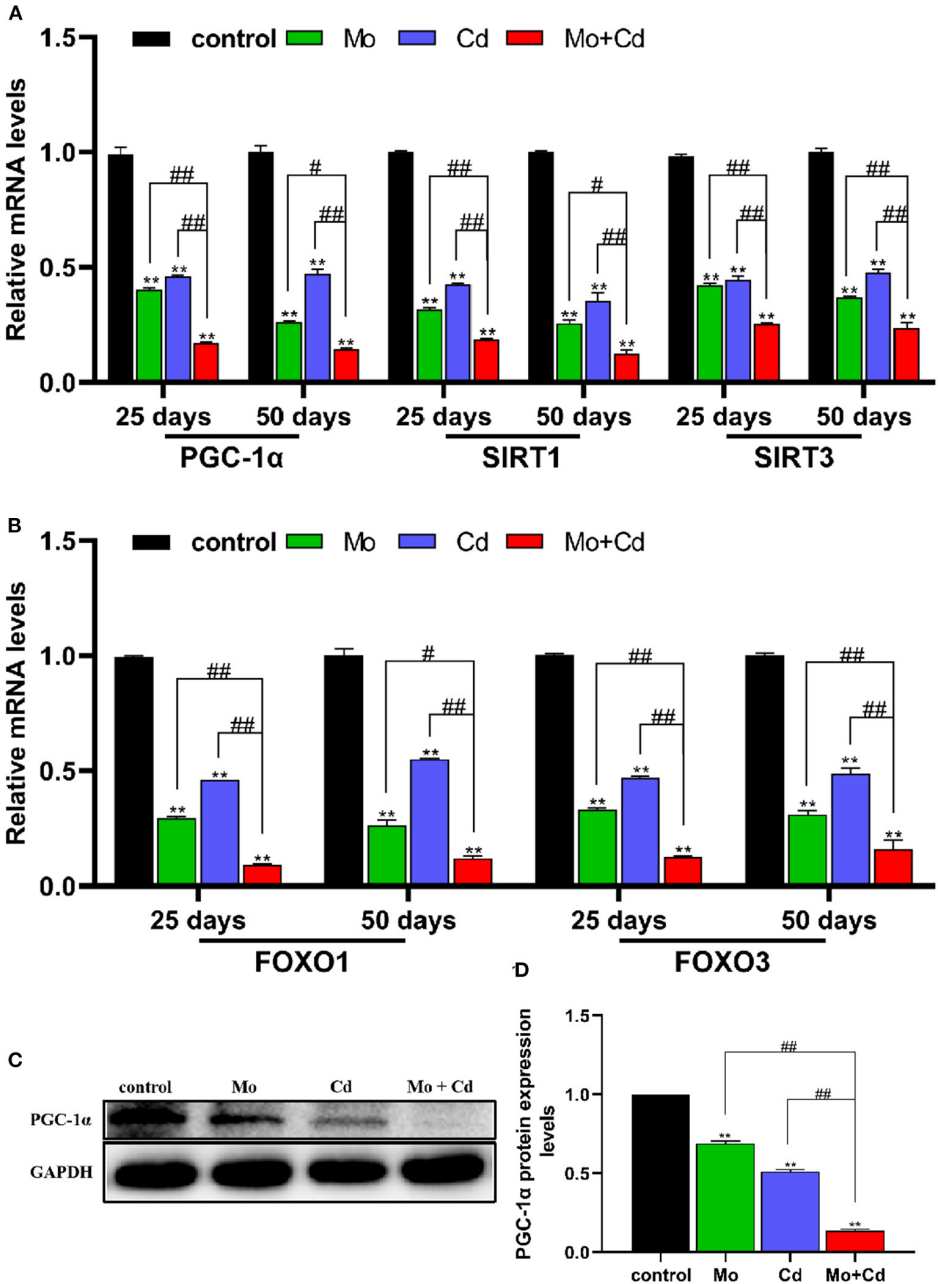
Mo or/and Cd exposure regulated mitochondrial biogenesis. **(A,B)** The mRNA levels of PGC-1α, SIRT1, SIRT3, FOXO1 and FOXO3. **(C)** The western blot results of PGC-1α. **(D)** The quantification of PGC-1α protein levels. Data are expressed as means ± SD (*n* = 6). “*” indicates significant difference compared to the corresponding control (*P < 0.05, **P < 0.01). “#” indicates statistically significant difference between corresponding groups (^#^*P* < 0.05, ^*##*^*P* < 0.01).

As shown in Figures 5A,B, Mo and/or Cd coerce significantly decreased (*P* < 0.05 or *P* < 0.01) the mRNA levels of mitochondrial biogenesis related genes (PGC-1α, SIRT1, SIRT3, FOXO1 and FOXO3) at 25 and 50 days. Moreover, we examined the protein levels of PGC-1α at day 50, it found that Mo and/or Cd treatment dramatically decreased the protein expression of PGC-1α (Figures 5C,D).

### Mo or/and Cd Exposure Induced FUNDC1-Mediated Mitophagy

**Figure 6:**
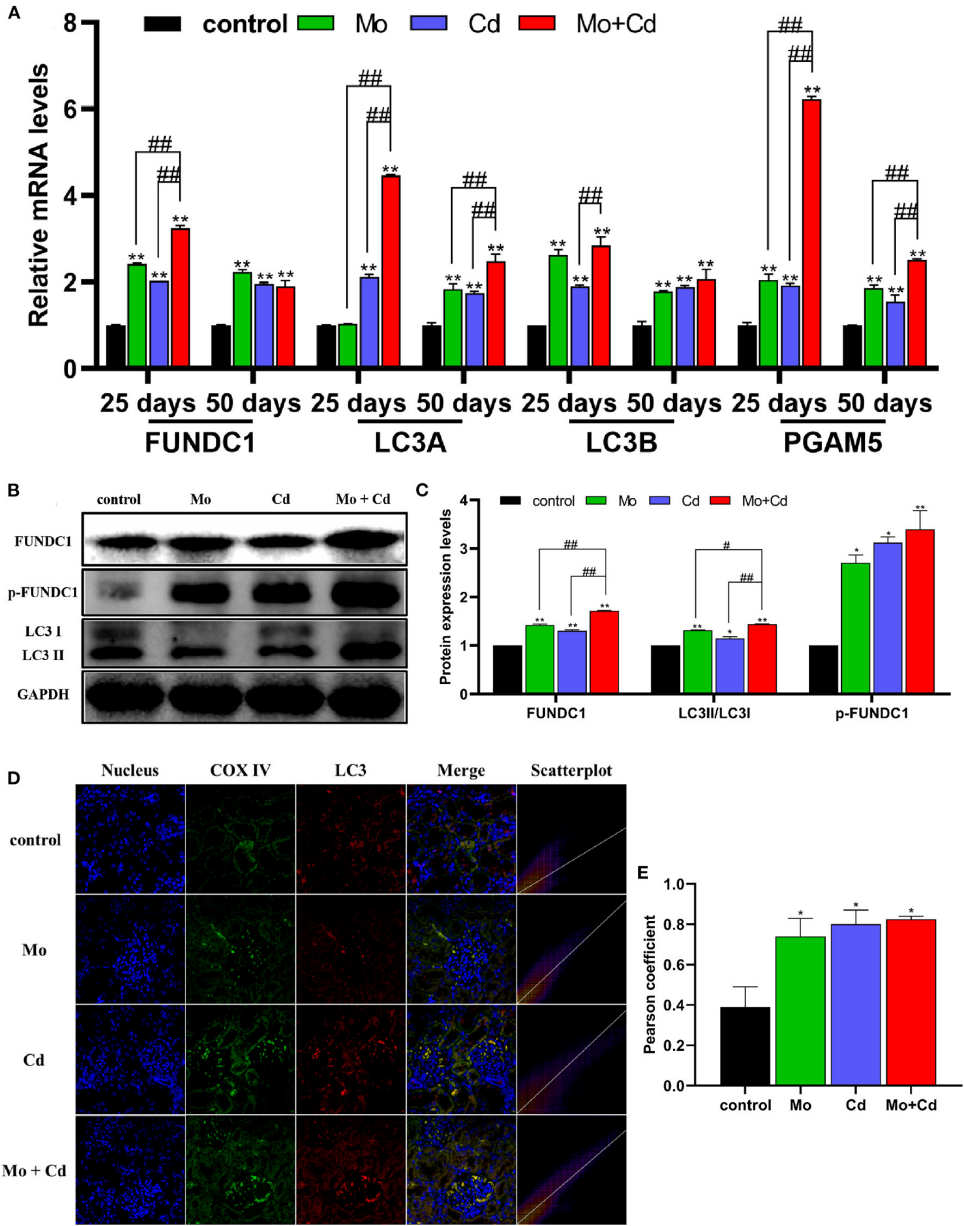
Effects of Mo and/or Cd on immunofluorescence of LC3, mitophagy-related mRNA and protein expression levels. **(A)** The mRNA levels of FUNDC1, LC3A, LC3B and PGAM5. **(B)** The western blot results of FUNDC1, p-FUNDC1 and LC3. **(C)** The quantification of FUNDC1, p-FUNDC1 (Ser17) and LC3II/LC3I protein levels. **(D)** Immunofluorescence co-location of COX IV and LC3 at day 50. In the images, the nucleus staining is shown in blue, COX IV staining is shown in green, LC3 staining is shown in red, and the signals of colocalization are shown in merged images. **(E)** Pearson coefficient of the colocalization of COX IV and LC3. Data are expressed as means ± SD (*n* = 6). “*” indicates significant difference compared to the corresponding control (*P < 0.05, **P < 0.01). “#” indicates statistically significant difference between corresponding groups (^#^*P* < 0.05, ^*##*^*P* < 0.01).

Figure 6A revealed that the mRNA levels of LC3A, LC3B, FUNDC1 and PGAM5 were upregulated especially (*P* < 0.01) at days 25 and 50 in all treatment groups than that in the control group. In addition, the protein levels of LC3II/LC3I, FUNDC1 and p-FUNDC1 increased (*P* < 0.05 or *P* < 0.01) markedly after Mo or/and Cd treatment at day 50 (Figures 6B,C), but the variation had distinct alteration in the joint group than those in single groups. The result of immunofluorescence co-location of COX IV and LC3 at day 50 is shown in Figures 6D,E. The green and red fluorescence signals overlapped in all treatment groups, which indicated that LC3 were recruited to mitochondria.

## Discussion

The kidney is a crucial metabolic organ of the body, which needs a lot of energy to actively maintain its metabolism. Mitochondria are particularly important in metabolically active organs such as the kidney, heart and liver (33). It has developed elaborate quality control systems to ensure the presence of the necessary number of functional mitochondria and to maintain a healthy and functioning mitochondrial network (19). Mitophagy reduces mitochondrial mass by removing damaged mitochondria, which is an important part of mitochondrial quality control. Previous studies have revealed that co-exposure of Mo and Cd can cause oxidative stress and mitophagy via the ROS-mediated PINK1/Parkin pathway (11, 16). However, whether Mo and Cd induce mitophagy through the FUNDC1 pathway has not been reported. It is well known that the kidney is the main target organ for Mo and Cd accumulating and poisoning. Due to the widespread presence of environmental pollution, the toxicology of Mo and Cd has been confirmed, but its potential mechanism remains unclear. Consequently, events leading to Mo and Cd co-induced mitochondrial quality control disorder via FUNDC1-mediated mitophagy in sheep kidney were experimented.

Excessive intake of Mo can cause obvious damage in many organs in function and morphology (9). Cd is a kind of heavy metal element, which is transmitted to animals and human beings through the food cycle, and accumulated in various organs in these places, causing damage to the body (34). In this study, the contents of Mo and Cd in the kidney were spotted. The results indicated that Mo and Cd cumulated in the kidney. Moreover, the contents of Cu and Zn in the kidney were significantly increased under Cd or/and Mo exposure. It is interesting to note that the accumulation of Mo and Cd in the kidney had a synergistic effect. Mo induced Cu deficiency is a common problem in ruminants. Mo combines with hydrogen sulfide in the rumen to form thiomolybdate, which prevents Cu from being absorbed, thus resulting in a decrease in Cu concentration in plasma. Conversely, high level of Mo could cause an increase in Cu content in the kidney (35). Residual Cu in plasma may constitute a non-available form of Cu that does not accumulate in the liver but is removed by the kidneys and eventually excreted in the urine (36). Exposure to Cd results in the production of renal metallothionein (MT), which binds Zn as well as Cd and therefore the concentration of Zn in the kidney increased with the increase of Cd concentration (37), which was consistent with our results. It is interesting to note renal MT may also bind Cu (38), which may explain the increase of Cu content in the kidney under Cd exposure. The kidney is known as the primary target of Mo and Cd poisoning (39). Mo or Cd accumulation could cause renal tubular epithelial injury, which is the main target site of renal injury (4). Our results showed that Mo or/and Cd treated sheep kidney tissue sections show renal lesions with granular and vacuolar degeneration of renal tubular epithelial cells. Numerous researches have displayed that exposure to heavy metals can give rise to oxidative stress *in vivo* (9, 12, 16, 31). The results showed that exposure to Cd or/and Mo significantly increased the contents of MDA and H_2_O_2_, which indicated that oxidative damage had occurred in the kidneys.

Next, a lot of mitophagosomes and vacuole-mitochondria were observed by transmission electron microscope. In eukaryotic cells, mitochondria are involved in energy generation, lipid metabolism and calcium homeostasis (26). We hypothesized that the morphological changes of mitochondria might lead to ATP production disturbance. ATP levels can be used to monitor the status of mitochondrial function, which is vital for healthy or diseased cells (40). Depletion of ATP synthesis leads to the use of most mitochondrial oxygen to produce ROS, generating an increase in cell lesion (11). Thus, we measured the content of ATP. The results displayed that the content of ATP in the kidney decreased significantly by Mo and/or Cd treatment. Therefore, mitochondrial morphology and function were impaired by exposure to Mo and Cd.

Mitochondria are dynamic organelles that constantly sustain fission and fusion, which are essential for cell survival and adaptation to changes in the cell growth, division and distribution of mitochondria (41). Mitochondrial fusion is mainly mediated by MFN1, MFN2 and OPA1. Mitochondrial fission is primarily mediated by DRP1 (42). MFN1 and MFN2 mediate the fusion of mitochondrial outer membrane, and OPA1 induces the fusion of mitochondrial inner membrane (43). Drp1, a key mediator of mitochondrial fission in the cytoplasm, is recruited to the outer membrane of mitochondria to promote mitochondrial fission by binding to receptor proteins, FIS1 (44). In addition, FIS1 and MFF overexpression induce mitochondrial fission (31). Mitochondria inhibit and delay the accumulation of abnormal changes through the fusion and fission of related proteases in organelles so as to maintain the normal operation of various physiological functions of mitochondria. Maladjustment of mitochondrial dynamics can lead to the decline of mitochondrial quality and abnormal mitochondrial movement (45). It was found that FUNDC1 interacts with OPA1 or DRP1 to regulate mitochondrial fission, fusion and mitophagy (20). In this research, the mRNA and protein levels of mitochondrial fusion factor and mitochondrial biosynthesis factor was limited, while the mRNA and protein levels of mitochondrial fission factor was raised under Mo and/or Cd pressure, leading to mitochondrial dynamics unbalance. PGC-1α is a primary regulator of mitochondrial biogenesis and function (46). SIRT1 and SIRT3 are crucial coordinators of PGC-1α, adjusting the biogenesis and function of the mitochondria (47). FOXO1 and FOXO3 bind to the PGC-1α promoter and enhance its transcription (48). We found that the Mo and/or Cd treatment observably decreased both the mRNA and protein levels of PGC-1α, and markedly reduced the mRNA levels of SIRT1, SIRT3, FOXO1 and FOXO3. Therefore, Mo or/and Cd exposure reduced mitochondrial mass by causing mitochondrial dynamics imbalance and blocking mitochondria biogenesis.

The specific quality control mechanisms of mitophagy play a crucial part in the clearance of dysfunctional and redundant mitochondria (49). The formation of mitophagosomes is the focus of course of mitophagy. In this research, the formation of mitophagosomes were observed by transmission electron microscope. It was found that Mo and/or Cd promoted the formation of mitophagosomes in sheep kidneys, and showed the synergistic relationship between Mo and Cd. Moreover, the transformation of LC3 from LC3I to LC3II is the pivotal step of mitophagosome formation, and LC3II content reflects straightway the amount of mitophagosomes (50). Cytochrome c oxidase (COX) subunits, complex IV in the mitochondrial respiratory chain, are located in the inner mitochondrial membrane (51). In our research, treatment with Mo and/or Cd enhanced the co-localization of COX IV-labeled mitochondria with LC3. In other words, an enhancement in LC3 puncta was spotted in mitochondria exposed to Mo and Cd. FUNDC 1 is a novel mitochondrial membrane protein that mediates mitophagy in mammalian cells (52), which could be phosphorylated at Ser17, leading to its enhanced interaction with LC3B for the activation of mitophagy (53). Our research found that the mRNA levels of LC3A, LC3B and FUNDC1 were markedly aggrandized at 25 and 50 days in all treatment groups than that in the control group. In addition, the protein levels of LC3II/LC3I, FUNDC1 and p-FUNDC1 (Ser17) were increased significantly under Mo or/and Cd pressure. Interestingly, we discovered that the mRNA level of PGAM5 was significantly upregulated by exposure to Mo or/and Cd. Previous studies have found that PGAM5 dephosphorylates Ser13, thereby promoting FUNDC1 interaction with LC3 and mitophagy (54). Therefore, our results revealed that Mo and/or Cd induce mitophagy through the FUNDC1 pathway.

In conclusion, Mo or/and Cd exposure might co-induce mitochondrial quality control disorder via FUNDC1-mediated mitophagy in sheep kidney, and Mo and Cd might display synergic interactions. However, the mechanism of mitochondrial quality control system in Mo and Cd induced nephrotoxicity remains to be further explored.

## Data Availability Statement

The original contributions presented in the study are included in the article/Supplementary Material, further inquiries can be directed to the corresponding author/s.

## Ethics Statement

The animal study was reviewed and approved by the Animal Ethics Committee of Jiangxi Agricultural University.

## Author Contributions

YW, FY, GZ, QW, CX, HB, XY, ZX, SY, and HC were responsibility for experiment conception, design, and practice. YW and FY were involved in the drafting of the manuscript. HC revised the manuscript. All authors contributed to the article and approved the submitted version.

## Funding

This work was supported by the program of Jiangxi Province Cattle and Sheep Disease Prevention and Control Position System (CARS-37), Jiangxi Province Education Department Science Foundation (No. GJJ14294), and Jiangxi Province Key Research and Development Project (No. 20192BBF60035).

## Conflict of Interest

The authors declare that the research was conducted in the absence of any commercial or financial relationships that could be construed as a potential conflict of interest.

## Publisher's Note

All claims expressed in this article are solely those of the authors and do not necessarily represent those of their affiliated organizations, or those of the publisher, the editors and the reviewers. Any product that may be evaluated in this article, or claim that may be made by its manufacturer, is not guaranteed or endorsed by the publisher.
