# Supplementary material for: Molybdenum and Cadmium Co-induce Mitochondrial Quality Control Disorder via FUNDC1-Mediated Mitophagy in Sheep Kidney
Source: Front Vet Sci. 2022 Jan 28;9:842259. doi: 10.3389/fvets.2022.842259 (PMC8831900; doi:10.3389/fvets.2022.842259)
Supplement: Supplementary file 1 [file Data_Sheet_1.doc]

**Table S1 Composition and nutrient levels in the basal diet for the sheep**

| Composition of diet | | Nutrient levels | |
| --- | --- | --- | --- |
| Ingredient | Content (%) | Index | level |
| Maize | 52.5 | CP (%) | 16.57 |
| Deoiled rice | 19.0 | ME (MJ kg^−1^) | 13.12 |
| Soybean meal | 10.0 | Ca (%) | 0.90 |
| Rapeseed meal | 7.0 | P (%) | 0.78 |
| Cottonseed | 7.0 |  |  |
| Limestone | 1.5 |  |  |
| CaHPO_4_ | 1.0 |  |  |
| Salt | 1.0 |  |  |
| Additives^1^ | 1.0 |  |  |
| Total | 100 |  |  |

^1^Per kilogram of additives contained the following: nicotinic acid 2000 mg, V_A_ 1,000,000 IU, V_D_ 3,250,000 IU, V_E_ 2400 mg, Zn (ZnSO4·H2O) 140,000 mg, Fe (FeSO4·H2O) 2000 mg, Mn (MnSO4·H2O) 3000 mg, I (KI, 3%) 180 mg, and Se (NaSe3O4·H2O) 100 mg

**Table S2** Abbreviations

| abbreviations | full till |
| --- | --- |
| FUNDC1 | FUN14 domain-containing 1 |
| MDA | malondialdehyde |
| H_2_O_2_ | hydrogen peroxide |
| ATP | adenosine triphosphate |
| LC3 | microtubule-associated protein 1 light chain |
| PGAM5 | Phosphoglycerate mutase 5 |
| DRP1 | dynamin-related protein 1 |
| FIS1 | mitochondrial adaptor fission 1 |
| MFF | mitochondrial fission factor |
| MFN1 | mitofusin 1 |
| MFN2 | mitofusin 2 |
| OPA1 | optic atrophy 1 |
| PGC-1α | peroxisome proliferator-activated receptor γ coactivator 1α |
| SIRT1 | sirtuin 1 |
| SIRT3 | Sirtuin 3 |
| FOXO1 | forkhead box O1 |
| FOXO3 | forkhead box O3 |
